# A role for the Hsp90 molecular chaperone family in antigen presentation to T lymphocytes via major histocompatibility complex class II molecules

**DOI:** 10.1002/eji.200535326

**Published:** 2006-04-01

**Authors:** Deepa Rajagopal, Vineeta Bal, Satyajit Mayor, Anna George, Satyajit Rath

**Affiliations:** 1 National Institute of Immunology, New Delhi, India; 2 National Centre for Biological Sciences, Bangalore, India

**Keywords:** Antigen presentation, Heat shock proteins, Macrophages, Major histocompatibility complex, T cell epitopes

## Abstract

The heat shock protein (HSP) Hsp90 is known to chaperone cytosolic peptides for MHC class I (MHCI)-restricted antigen presentation toT lymphocytes. We now demonstrate a role for Hsp90 activity in presentation of antigens on MHCII. Treatment of mouse antigen-presenting cells (APC) with the pharmacological Hsp90 inhibitor, geldanamycin, inhibited MHCII-mediated presentation of endocytosed and cytosolic proteins as well as synthetic peptides to specific T cells. Ectopic expression of human Hsp90 in APC enhanced MHCII-mediated antigen presentation. Further, pharmacological Hsp90 inhibition reduced, while retroviral Hsp90 overexpression enhanced, the levels of stable compact MHCII heterodimers correlating with the antigen presentation phenotype. Pharmacological inhibition of Hsp90 activity in IFN-γ-treated APC resulted in severe abrogation of MHCII-restricted presentation of cytosolic antigen, but only partially inhibited exogenous antigen presentation. Our data suggest a major role for Hsp90 activity in MHCII-mediated antigen presentation pathways, and implicate IFN-γ-inducible Hsp90-independent mechanisms.

## Abbreviations

MHCI: MHC class I
MHCII: MHC class II

## Introduction

The molecular chaperone Hsp90 is critical to a multitude of cellular functions [1, 2]. Hsp90 can act as a general chaperone preventing the aggregation of unfolded polypeptides [3, 4] and, in concert with chaperones of the Hsp70 family and other co-chaperones, is intimately associated to protein degradation in the cell. Hsp90 has been described as the cellular target of benzoquinoid ansamycin drugs such as geldanamycin and herbimycin [5] shown to prevent Hsp90-mediated refolding of a variety of cellular proteins, directing them for degradation by the proteasome. X-ray crystallo-graphic evidence suggests that geldanamycin serves as an ATP/ADP mimetic and binds the amino-terminal pocket of Hsp90 [6], inhibiting ATPase activity and consequently ATP-dependent peptide release [7]. Association of Hsp90 with the 20S proteasome has been shown to influence proteasomal enzymatic activity [8].

Loading of peptides generated in APC such as macrophages and B lymphocytes on MHC molecules is essential for recognition by T lymphocytes. HSP-chaperoned peptides are loaded on to MHC class I (MHCI) with much greater efficiency than free peptides in the cytosol [9], which may otherwise be prone to cytoplasmic proteolysis in the absence of chaperone association. Recent evidence demonstrates the role of HSP in cross priming of CD8 T cells, which recognize their target peptides bound to MHCI [10].

While cytosolically generated ligands are loaded on MHCI in the endoplasmic reticulum [11], MHC class II (MHCII) molecules bind to peptides in endolysosomal compartments, which are then presented to CD4 T cells. Assembled MHCII αβ heterodimers traverse from the ER to the late endocytic compartments bound by the invariant chain (Ii) [12, 13]. Endolysosomal cysteine proteinases or cathepsins [14] mediate steps of Ii degradation in APC leaving a short MHCII-associated Ii peptide, CLIP, protecting the peptide-binding groove. The H-2 M molecule facilitates CLIP removal [15], rendering the MHCII molecule capable of peptide binding and stable MHCII-peptide complexes transit to the plasma membrane for T cell recognition.

Peptides from cytoplasmic antigens have also been shown to gain access to the MHCII pathway for presentation [16–20]. Ligand generation through alternate pathways complements conventional processing events and widens the spectrum of epitopes made available for recognition by CD4 Tcells [19, 20]. We have previously reported that processing events generating MHCII-binding peptides in the cytosol are distinct from those generating peptides in the endocytic pathway and that endogenous processing is reliant on proteasomal function [18]. Our data have further demonstrated that endolysosomal vesicular compartments are a point of convergence for peptides generated both by endolysosomal as well as by cytosolic processing events [21]. Distinct proteolytic mechanisms operative in different APC subpopulations and in different subcellular locations may create quantitative and qualitative alterations in the nature of peptides complexed to MHC and as a result the T cell response [22]. Putative peptide ligands are constantly faced with the constitutive proteolytic machinery of the cell that in the process of epitope generation could incur epitope destruction. Peptide epitope precursors have been found to be associated *in vivo* with heat shock proteins such Hsp70, Hsp90 and the ER chaperone, gp96, providing a mechanism for HSP to modulate antigen presentation [23].

Most studies thus far have focused on the interactive role of chaperones with components of the MHCI pathway [24, 25]. Experimental studies also provide evidence for HSP-peptide complexes in facilitating in vivo presentation of MHCII-restricted epitopes [25–27].

As HSP proteins associate *in vivo* with a wide range of intracellular peptides generated in varied sub cellular locations, we asked whether the presentation of endosomally or cytosolically generated MHCII ligand peptides is modulated byHsp90. Our data demonstrate a requirement for Hsp90 in MHCII-mediated presentation of peptides from all sources. We suggest that Hsp90 may modulate antigen presentation in part by effects on stable MHCII heterodimer formation or persistence. Further, the requirement for Hsp90 functions in antigen presentation differs between cell lineages for MHCI and can be partially bypassed by IFN-γ activation of APC for MHCII.

## Results

### General remarks

The finding that a class of ansamycin antibiotics such as geldanamycin specifically bind Hsp90 [5–7] allowed us to begin addressing the specific role of Hsp90 in antigen presentation pathways.

### Geldanamycin inhibits MHCII-restricted presentation by antigen-loaded B cell and macrophage APC

**Figure 1:**
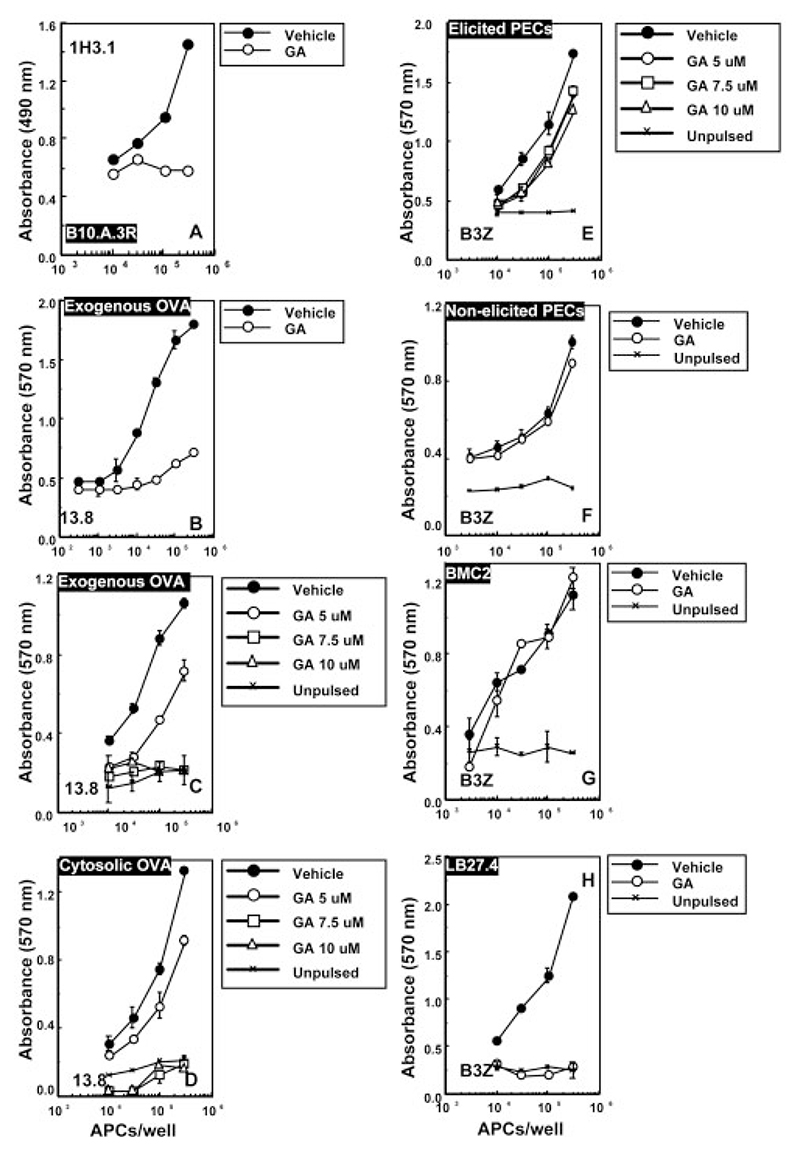
Geldanamycin inhibits MHCII-restricted presentation of endogenous and exogenous proteins. (A, B) Peritoneal macrophages elicited from B10.A(3R) mice, treated for 16 h with either geldanamycin (GA; 5 μM) or vehicle alone, were pulsed exogenously with OVA (3 mg/mL) in the medium for 3 h in the presence or absence of GA, fixed, washed and assessed for either endogenous EapL presentation to the 1H3.1 T cell line (A), or exogenous OVA presentation to the 13.8 T cell line (B). (C−E) Peritoneal macrophages elicited from C57BL/6 mice, treated for 1 h with titrating concentrations of GA, were loaded with OVA (10 mg/mL) either exogenously (C) or cytosolically (D, E) as indicated. The cells were incubated for an additional 3 h in the presence or absence of GA, washed, fixed and used as APC for presentation to 13.8 (C, D) or B3Z (E) T cells as indicated. Unpulsed APC were used as negative controls. (F−H) Resting peritoneal macrophages from C57BL/6 mice (F), BMC-2 cells (G) or LB27.4 cells (H) treated for 1 h with either 7.5 μM GA or vehicle, were loaded cytosolically with OVA (10 mg/mL). The cells were incubated for an additional 3 h in the presence or absence of GA, washed, fixed and used as APC for presentation to B3Z T cells as indicated. Unpulsed APC were used as negative controls. In all panels, the IL-2 (1H3.1) or β-galactosidase (13.8 and B3Z) levels induced are indicated, and all data are representative of at least three independent experiments. Error bars represent mean ± standard deviation of triplicate wells.

We studied the presentation of a well-characterized APC-endogenous peptide for its sensitivity to geldanamycin. Processing of the extracytoplasmic region of the MHCII polypeptide I-Eα (H2-Eα) yields an epitope, EapL (aa 52−68), which binds to and is presented in the context of H2-A^b^. APC from mouse strains such as B10.A(3R) constitutively express the Eap-H2-A^b^ complex on their surface [28]. We used peritoneal macrophages from B10.A(3R) mice to study the effects of geldanamycin on endogenous presentation of EapL-H2-A^b^ to the Tcell hybridoma 1H3.1, which recognizes both the peptide from derived from exogenous pulsed protein sources, EapS (aa 52−66), as well as the constitutive endogenous derived long peptide, EapL (aa 52−68), in the context of H2-A^b^. Geldanamycin completely inhibited presentation of endogenous EapL to 1H3.1 (Fig. 1A). Further, MHCII-restricted presentation of exogenous OVA by the same APC to the Tcell hybridoma 13.8, was similarly abolished (Fig. 1B). Similar results were obtained when the B cell line LB27.4, which also expresses H2-Eα constitutively, was used as APC (data not shown).

Recent studies have demonstrated the effect of bacterially derived HSP on the exogenous antigen-processing pathway for MHCII [29]. Exogenous antigen entry to the MHCII-mediated presentation pathway occurs via endocytosis or fluid phase uptake by pinocytosis and contributes to the better-characterized conventional pathway for internalization and presentation of extracellular ligands on MHCII. As a complementary approach for antigen delivery directly to the cytosol, we have made use of osmotic lysis of pinosomes, which has been previously shown to introduce exogenous antigens into proteasome-dependent pathways of antigen presentation [18, 30]. This methodology allows for delivery and parallel assessment of equivalent quantities of the same protein in distinct subcellular locations. Using such a system, we looked at effects of geldanamycin on MHCII-restricted presentation of endocytosed versus cytosolically introduced protein.

Peritoneal macrophages were pulsed with OVA either through the exogenous pathway by pinocytosis or directed to the cytosol by osmotic lysis of pinosomes. Mock-loaded or antigen-loaded macrophages were used as APC. While APC loaded with antigen through either mode of delivery presented OVA efficiently on MHCII to 13.8 cells, presentation was inhibited in a dosedependent manner by geldanamycin (Fig. 1C and D). We assessed treated APC by trypan blue exclusion and found no adverse effects of geldanamycin on cell viability. Since a concentration of 7.5 μM geldanamycin was found to effectively inhibit MHCII-restricted antigen presentation with no adverse effects on cell viability, we used this concentration for treatment of APC prior to and during antigen loading in all further assays.

Interestingly, the same macrophage APC showed no significant inhibition when assessed for presentation of cytosolically loaded OVA to the K^b^-restricted T cell line, B3Z (Fig. 1E). Since these experiments used thiogly-collate-activated peritoneal macrophages as APC, it was imperative to ascertain the contribution of inherent activation status of primed APC to the observed pattern of presentation. To address this, we used either non-activated, peritoneal resident cell-derived macrophages (Fig. 1F), or the H-2^b^ BMC-2 monocytic cell line (Fig. 1G) as APC. In these monocytic APC, too, MHCI-restricted presentation of cytosolic OVA was not inhibited by geldanamycin (Fig. 1F and G). These results indicate that the geldanamycin-insensitivity of cytosolic OVA presentation on MHCI is independent of maturation status of the macrophage APC. While ruling out the possible toxic effects of the drug, these results present an apparent discordance with earlier data that support a role for Hsp90 function in MHCI-restricted antigen presentation [31]. A potential explanation came from the observation that MHCI-restricted cytosolic OVA presentation by splenic mixed APC populations was sensitive to geldanamycin (data not shown), indicating possible lineage-specificity in the Hsp90 requirement for the MHCI presentation pathway. We directly addressed this possibility by assessing the geldanamycin sensitivity of the presentation of cytosolically delivered OVA in the B cell line LB27.4. Unlike the monocytic APC, MHCI-restricted cytosolic OVA presentation by LB27.4 was geldanamycin sensitive (Fig. 1H).

Together, the above data show that while MHCII-restricted presentation of both exogenous and cytosolically delivered antigen as well as of constitutively expressed endogenous antigen is uniformly geldanamycin sensitive, there exist APC lineage-specific differences in the effects of geldanamycin on the MHCI-mediated antigen presentation pathway.

### Peptide presentation on MHCII but not MHCI is geldanamycin sensitive

**Figure 2:**
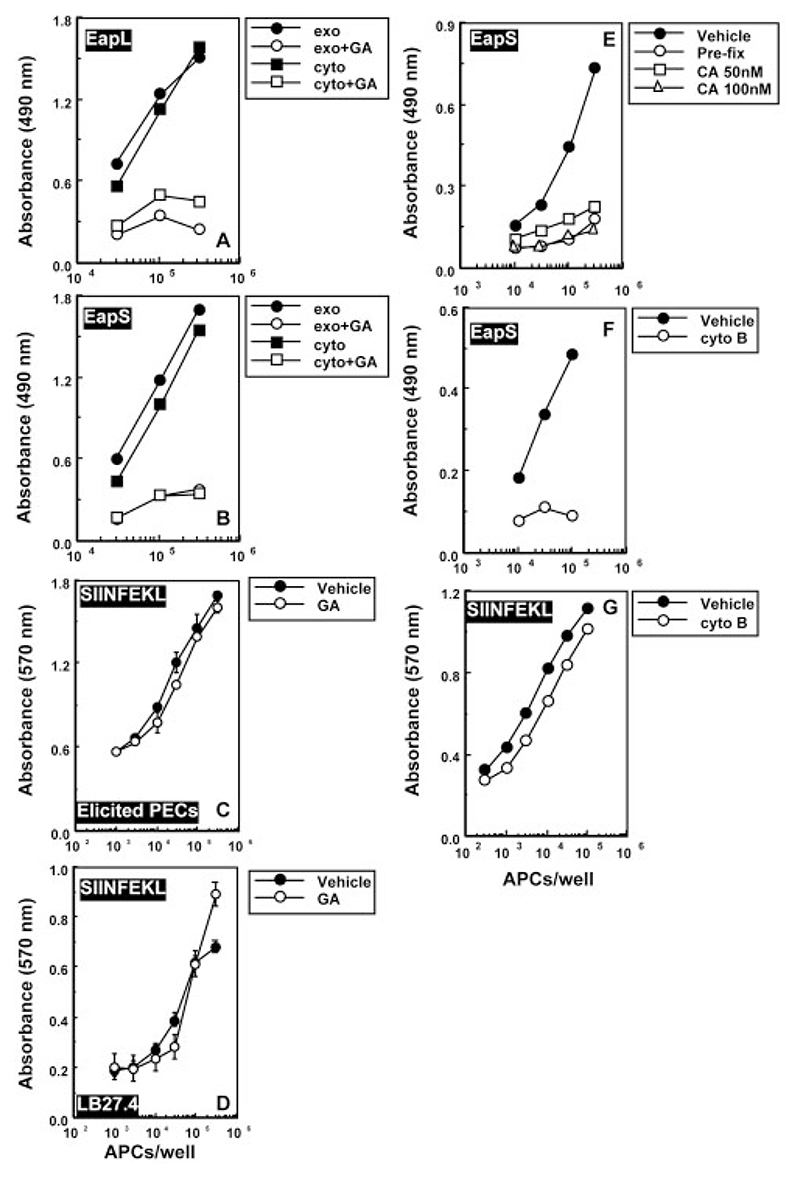
Geldanamycin inhibits MHCII-restricted peptide presentation. (A−C) Peritoneal macrophage APC elicited from C57BL/6 mice, or LB27.4 cells (D), treated with either GA (7.5 μM) or vehicle alone were pulsed exogenously (exo) or cytosolically (cyto) with EapL (A) or EapS (B), or with exogenous SIINFEKL peptide (C, D), and responses of the MHCII-restricted T cell line 1H3.1 (A, B) or of the MHCI-restricted line B3Z (C, D) were tested. (E, F) C57BL/6 macrophage APC were either pre-fixed with 0.1% paraformaldehyde (E; ‘pre-fix’) or treated with the indicated concentrations of concanamycin A, CA, (E) or were treated (F) with cytochalasin B, cyto B, prior to EapS pulsing and use for assessing the responses of the 1H3.1 T cell line. Cytochalasin B-treated APC were also pulsed with the SIINFEKL peptide and assessed for the ability to stimulate the MHCI-restricted B3Z T cell line (G). In all cases with inhibitor treatment, APC pretreated with vehicle alone were pulsed with the relevant peptide and used as controls. The data shown are representative of three to four independent experiments and error bars represent mean ± standard deviation of triplicate wells.

Inhibition of antigen presentation could either be due to effects on peptide generation or peptide loading. To further understand the step at which geldanamycin intercepts the MHCII pathway we looked at effects on presentation of synthetic peptides. Exogenous processing of the H2-Eα protein generates the short peptide EapS (aa 52−66), while constitutive processing of the endogenous protein leads to the generation of the long peptide EapL (aa 52−68) for presentation on H2-A^b^ [28]. Synthetic EapL or EapS were directly introduced into the cytosol or pulsed exogenously on macrophage APC preincubated with geldanamycin for 1 h prior to peptide loading. Presentation of both peptides on MHCII was completely blocked by geldanamycin (Fig. 2A and B). Under similar conditions of treatment, exogenous presentation of the K^b^-restricted OVA-derived peptide SIINFEKL to the MHCI-restricted T cell line B3Z was unaltered (Fig. 2C). Since MHCI-restricted antigen presentation of both native antigen as well as peptide in macrophage APC appeared to be geldanamycin resistant, we also examined the geldanamycin sensitivity of SIINFEKL presentation by the B cell line LB27.4, wherein the MHCI-mediated OVA presentation is geldanamycin sensitive. SIINFEKL presentation by these B cell APC was found unaltered by geldanamycin treatment (Fig. 2D), indicating that the inhibitory effects of geldanamycin on peptide presentation were specific to MHCII. The difference in pattern of geldanamycin sensitivity of peptide loading on MHCI versus MHCII was further assessed by looking into the requirement of metabolically active APC for EapS presentation using the following three approaches. First, paraformaldehyde fixation of APC prior to peptide pulse abrogated EapS presentation (Fig. 2E), suggesting that unlike MHCI-restricted presentation of SIINFEKL peptide, EapS presentation was not mediated by peptide loading at the cell surface and the process required metabolically active APC. Secondly, pretreatment of APC with an inhibitor of actin polymerization, cytochalasin B [32], resulted in a complete loss of EapS presentation (Fig. 2F), again suggesting peptide internalization is a pre-requisite for EapS presentation. Thirdly, treatment of APC with the vacuolar ATPase inhibitor, concanamycin A [33] that prevents endosomal acidification also abrogated EapS presentation (Fig. 2E). Thus, EapS presentation depends on internalization and requires intact endosomal function. In all these experiments, no discernable effects on presentation of the SIINFEKL peptide (Fig. 2G and data not shown) were observed.

These results indicate that geldanamycin-mediated inhibition of peptide presentation is attributable at least in part to effects on endosomal post-processing peptide loading on MHCII rather than on peptide exchange at the cell surface.

### Overexpression of Hsp90 enhances antigen presentation

The effects of geldanamycin are reported to be mediated through its action as an Hsp90-specific inhibitor. The Hsp90 family includes the ER-resident glucose-regulated protein (Grp94) and the mitochondrial tumor necrosis factor receptor-associated protein (TRAP1) along with Hsp90 [34, 35]. To correlate the effects of geldanamycin with Hsp90, we made use of a complementary approach. Ectopic expression of either Hsp90 or enhanced green fluorescent protein (eGFP) was achieved in the H2-A^b^ monocytic cell line BMC-2 as well as in the B cell line LB27.4 by retroviral transduction. The transduced cells were grown under selecting conditions, and the resultant cell lines were used as APC for antigen-presentation assays.

**Figure 3:**
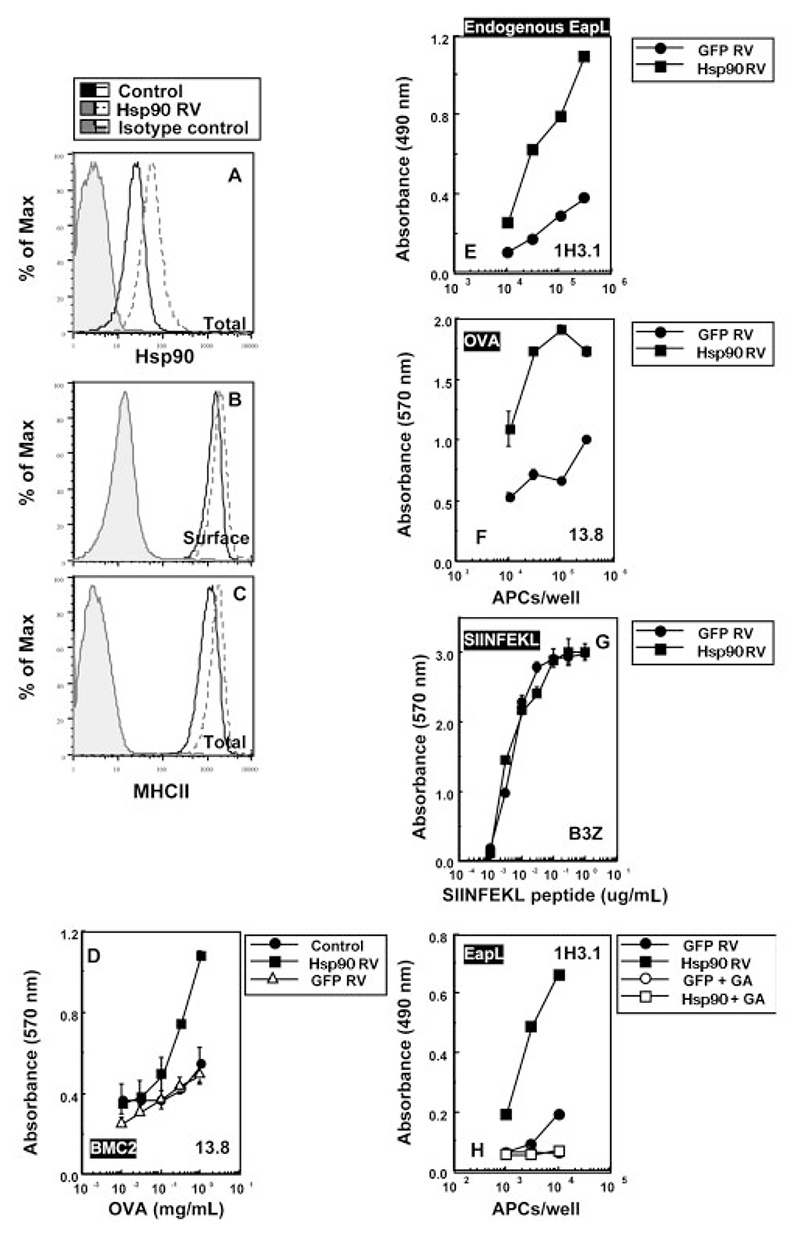
Ectopic expression of Hsp90 enhances MHCII-restricted antigen presentation. (A−C) Human Hsp90 was retrovirally transduced into the mouse B cell line LB27.4 and puromycin-selected cell populations tested for cellular expression of Hsp90 (A) and MHCII (B, C) in unpermeabilized for surface levels (B) and post-permeabilization for total levels on cells (C). The shaded histogram depicts the isotype control. (D) Untransduced control or retrovirally (Hsp90 or eGFP-RV) transduced BMC-2 cells selected for expression of human Hsp90 or eGFP were washed extensively and tested for presentation of pulsed exogenous OVA in graded doses to the MHCII-restricted T hybridoma 13.8. (E−G) Retrovirally transduced LB27.4 selected for carriage of human Hsp90 (Hsp90 RV) or eGFP (eGFP RV) were washed extensively and tested for endogenously derived EapL presentation to 1H3.1 (E), presentation of pulsed exogenous OVA (5 mg/mL) to the MHCII-restricted T hybridoma 13.8 (F) or presentation of exogenously pulsed SIINFEKL peptide (1 μg/mL) to the MHCI-restricted T cell line B3Z (G). (H) Control (eGFP) or Hsp90-expressing LB27.4 cells selected on puromycin were washed extensively and treated with 5 μM GA for 16 h, and tested for endogenous presentation of EapL to the T cell line 1H3.1. All data shown are representative of at least three independent experiments. Error bars represent mean ± standard deviation of triplicate wells.

Hsp90 transduction resulted in an increment in cellular Hsp90 expression as evidenced by FACS analysis (Fig. 3A), while total as well as surface MHCII levels remained relatively unaltered (Fig. 3B, C). We next assessed the antigen presentation ability of Hsp90-overexpressing cells and found that MHCII-restricted presentation of exogenous OVA by BMC-2 cells over-expressing Hsp90 was greatly enhanced as compared to untransduced or eGFP-transduced controls (Fig. 3D). An enhancement of endogenous H2-Eα-derived peptide presentation on MHCII was similarly observed in the B cell line LB27.4 overexpressing Hsp90 (Fig. 3E). Additionally, when these APC were pulsed with exogenous OVA, MHCII-restricted OVA presentation was also greatly enhanced (Fig. 3F). APC overexpressing Hsp90 however had unchanged potential to present the MHCI-restricted SIINFEKL peptide (Fig. 3G). Unaltered patterns of presentation in eGFP-transduced cells provided an appropriate control for possible incidental effects of retroviral transduction. In order to confirm further that the enhanced MHCII-restricted antigen presentation seen in Hsp90-transduced cells was attributable specifically to Hsp90 and not due to the inadvertent selection of an improved presentation phenotype, we examined the geldanamycin sensitivity of these Hsp90 overexpressing cells. Geldanamycin treatment resulted in complete loss of endogenous EapL presentation on MHCII in Hsp90-overexpressing LB27.4 (Fig. 3I). Taken together, the results suggest that Hsp90 is the geldanamycin target instrumental in modulating MHCII-restricted antigen presentation.

### Hsp90 affects formation of SDS-stable MHCII dimers

Results described thus far are suggestive of a role for Hsp90 in endosomal peptide loading on MHCII. An outcome of successful peptide loading is the generation of SDS-stable MHCII heterodimers [36, 37]. Surface and total cellular MHCII levels as observed in unpermeabilized and permeabilized APC, respectively, were found to remain unaffected by Hsp90 overexpression (Fig. 3C and D). Therefore, the enhanced presentation cannot be attributed to simple alterations in MHCII levels. The observed differences in MHCII presentation on geldanamycin treatment may thus indicate a possible endosomal function for Hsp90 that, by supporting efficient peptide loading, would modulate the formation and/or persistence of SDS-stable MHCII heterodimers. To assess this possibility, whole-cell lysates from Hsp90-transduced or eGFP-transduced LB27.4 were subjected to non-reducing SDS-PAGE followed by Western blot analysis using the H2-A^b^-specific mAb, Y3P.

**Figure 4:**
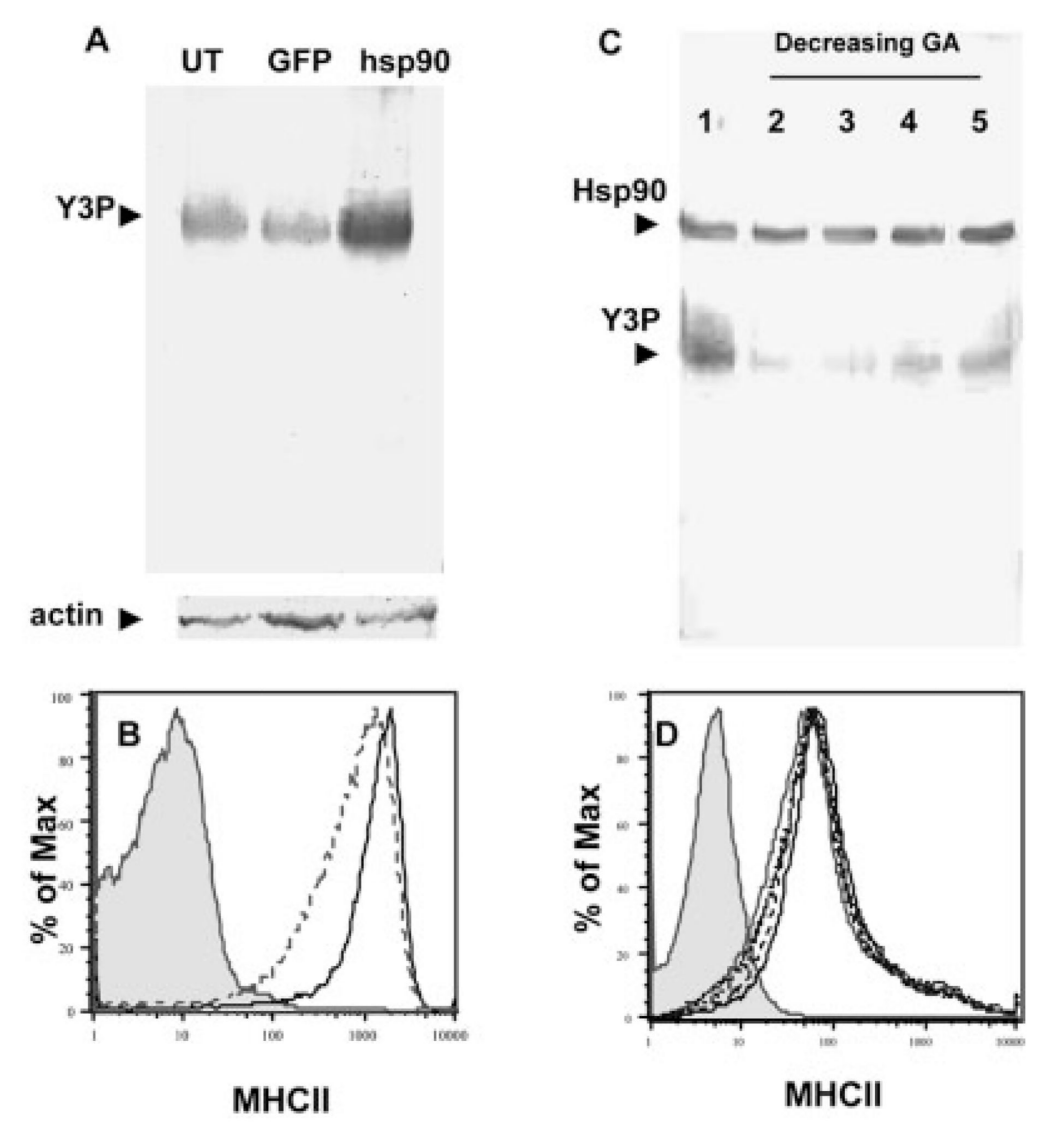
Hsp90 enhances SDS-stable MHCII heterodimer levels. (A) Whole-cell lysates from Hsp90-overexpressing LB27.4 cells were resolved on non-reducing SDS-PAGE and Western blotted with either the anti-I-A^b^ mAb, Y-3P or an antiactin antibody. The lanes indicate lysates from untransduced (1), control eGFP-retrovirus-transduced (2) and Hsp90 retrovirus-transduced (3) LB27.4 cells. (B) Surface MHCII levels in LB27.4 cells transduced with a control retrovirus (solid lines) or the Hsp90-bearing retrovirus (dotted lines). Shaded histogram represents isotype control. (C) Western blot analyses of LB27.4 whole-cell lysates left untreated (lane 1) or treated with various concentrations of geldanamycin (lanes 2−5; 10, 7.5, 5 and 2.5 μM, respectively) simultaneously probed with an anti-Hsp90 mAb and the anti-I-A^b^ mAb Y3P are shown. (D) Surface levels of MHCII are shown corresponding to lanes 1 to 5 in panel (C). All data shown are representative of two to four independent experiments.

LB 27.4 cells overexpressing Hsp90 revealed enhanced levels of SDS-stable MHCII heterodimers (Fig. 4A), while total MHCII levels were unaltered (Fig. 4B). The H2-A^b^-specific mAb Y3P does not recognize isolated MHCII polypeptides and therefore the SDS-unstable components of H2-A^b^ would not be visualized in such a Western blot analysis. Enhanced levels of SDS-stable MHCII heterodimers thus correlate with enhanced antigen presentation on MHCII (Fig. 3D−F). We also assessed the levels of SDS-stable MHCII dimers in geldanamycin-treated macrophage APC, which show an inhibition of MHCII-restricted antigen presentation. Treatment of C57BL/6 peritoneal macrophages with graded doses of geldanamycin prior to Western blot analysis revealed a reduction of SDS-stable MHCII heterodimers in a graded dose-response manner (Fig. 4C). However, both Hsp90 expression and total MHCII levels remained unchanged at all concentrations tested (Fig. 4D).

Thus, the loss or enhancement in antigen presentation on MHCII in geldanamycin-treated or Hsp90-overexpressing APC, respectively, correlates well with the generation of SDS-stable MHCII heterodimers. The unaltered surface and cellular MHCII levels rule out the possible effects of geldanamycin in MHCII biosynthesis. It seems plausible that the surface MHCII molecules in geldanamycin-treated APC may either be ‘floppy’ dimers that are empty or CLIP-bound [38].

### Effects of geldanamycin on MHCII-restricted antigen presentation in activated macrophage APC

**Figure 5:**
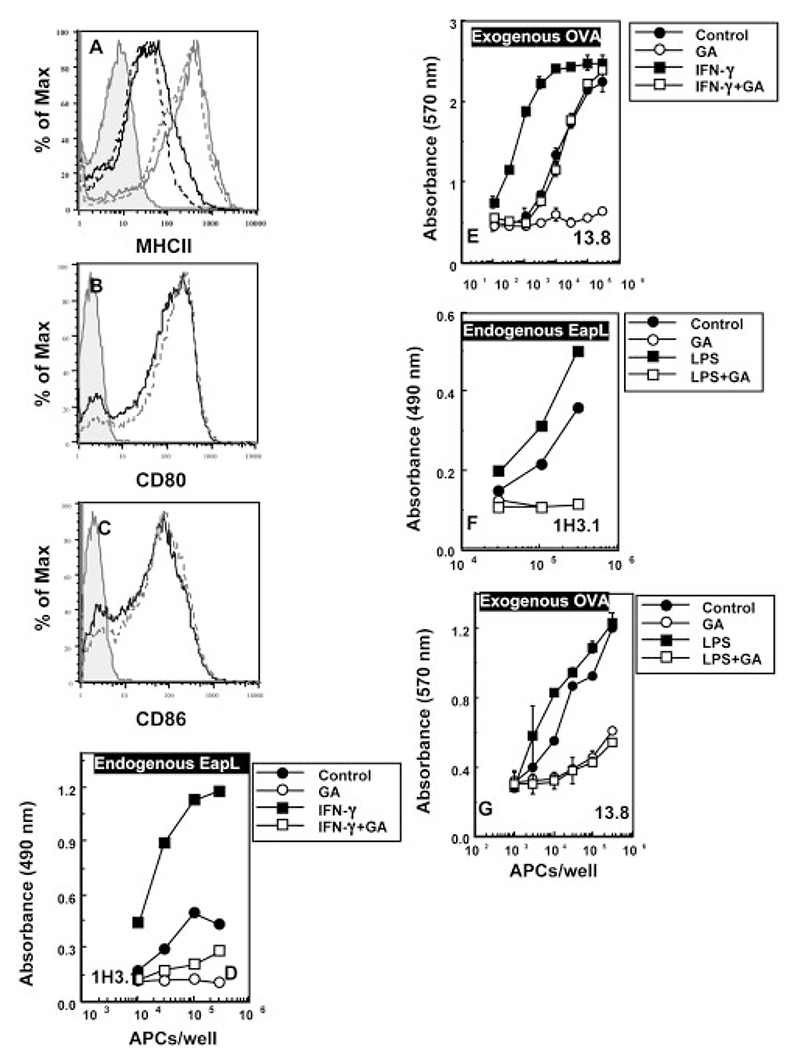
Geldanamycin affects MHCII-restricted antigen presentation by activated macrophage APC. (A) Peritoneal macrophages elicited from B10.A(3R) mice were rested overnight prior to stimulation with IFN-γ (gray lines) or left unstimulated (black lines). Both sets were either treated with GA (dotted lines) or left untreated (solid lines), were stained for surface MHCII expression and assessed by flow cytometry. (B, C) Surface levels of CD80 (B) and CD86 (C) expression on B10.A(3R) peritoneal macrophages either treated with GA (dotted lines) or left untreated (solid lines). (D, E) B10.A(3R) peritoneal macrophages as in panel (A), were either stimulated with IFN-γ or left unstimulated (control) for 48 h. During the last 16 h, the cells were treated either with geldanamycin (5 μM) or with vehicle alone. All cell groups were pulsed with 3 mg/mL OVA for the last 3 h of culture prior to fixation and use as APC for presentation to 1H3.1 (D) or 13.8 (E) T cell lines. (F, G) B10.A(3R) peritoneal macrophages were either stimulated with LPS or left unstimulated (control) for 48 h. During the last 16 h, the cells were treated either with geldanamycin (5 μM) or with vehicle alone. All cell groups were pulsed with 3 mg/mL OVA for the last 3 h of culture prior to fixation and use as APC for presentation to 1H3.1 (F) or 13.8 (G) T cell lines. All data shown are representative of two to three independent experiments.

Antigen presentation pathways for both MHCI and MHCII are affected by IFN-γ, which is known to induce MHC levels and cytosolic proteolytic machinery [39] as well as modulate endolysosomal protease activity [40, 41]. To examine the effect of IFN-γ activation on the role of Hsp90 in antigen presentation, we used IFN-γ-stimulated B10.A(3R)-derived macrophages. As noted above, APC from this recombinant mouse strain constitutively express the H2-Eα protein as well as the H2-A^b^ restricting element and therefore provide a system to assess effects on both endogenous and exogenous antigen presentation on MHCII by similarly treated APC. IFN-γ-activated APC revealed an increase in the surface levels of MHCII, which is unaffected by geldanamycin treatment (Fig. 5A). To exclude possible confounding influences of geldanamycin on other cell surface molecules, the expression of costimulatory molecules CD80 and CD86 was examined. As shown, the expression of CD80 (Fig. 5B) and CD86 (Fig. 5C) on similarly treated macrophage APC was unaffected by geldanamycin treatment.

MHCII-mediated presentation of endogenous H2-Eα as well as of exogenously delivered OVA were enhanced following IFN-γ-stimulation (Fig. 5D and E). Treatment with geldanamycin resulted in almost complete abrogation of MHCII-restricted endogenous H2-Eα presentation on both control and IFN-γ-stimulated macrophages (Fig. 5D). When these APC were pulsed exogenously with OVA during the last 3 h of incubation and MHCII-restricted OVA presentation assessed, we found that exogenous OVA presentation by resting APC was almost completely inhibited. In contrast, IFN-γ-activated APC retained a substantial capability to present exogenous OVA on MHCII even when treated with geldanamycin (Fig. 5E). Thus, in IFN-γ-activated APC, geldanamycin treatment led to a reduction of endogenous H2-Eα presentation by a dose-response curve shift of over 30-fold, while the reduction of exogenous OVA presentation by the same APC was only about 10-fold. These results indicate that a component of MHCII-restricted antigen presentation of exogenous antigen in IFN-γ-activated macrophage APC may be differentially dependent on Hsp90, while this is not the case for presentation of endogenous antigen. We observed no alteration of Hsp90 expression in activated APC (data not shown).

We further looked at the effects of APC activation by the microbial byproduct LPS on the Hsp90-dependence of MHCII-restricted presentation. Similar to the effects of IFN-γ, macrophage APC activated with LPS showed enhanced levels of surface MHCII that was unaltered by geldanamycin treatment (data not shown). Endogenous antigen presentation was found to remain sensitive to inhibition by geldanamycin (Fig. 5F). Interestingly, we found that, unlike the situation with IFN-γ treatment, the exogenous pathway of MHCII-mediated presentation also remained completely sensitive to geldanamycin in LPS-activated APC (Fig. 5G). These data suggest that the geldanamycin-insensitivity of the exogenous MHCII presentation pathway induced by IFN-γ is specific and is not mimicked by other pathways of APC activation.

## Discussion

Recognition of a broad spectrum of epitopes is crucial to the initiation of effective T cell responses and the ability of the immune system to cope with disparate site(s) of antigen access. We have been investigating pathways leading to MHCII-restricted presentation of ligands generated from endogenous antigen, conventionally recognized for presentation by the MHCI pathway. Data from others and our group envisage a dichotomy in the sub cellular location of antigens sampled and recognized by the MHCII presentation pathway [16–21]. Dependence on chaperone protection is well established for putative MHCI ligands and the ability of HSP such as Hsp70 and ER-Gp96 to co-ordinate binding and release of intracellular peptides in an ATP-dependent fashion argues for the notion that peptide ligands for MHCI binding may not simply diffuse to transporters but may in fact be chaperoned [10, 42, 43].

Fluid-phase endocytosis allows for antigen access to the conventional route for processing and presentation through the MHCII pathway. Analysis of peptides on MHCII however reveals many MHCII-binding peptides to be from cellular rather than extracellular protein sources [44, 45]. We have made use of hyperosmotic lysis of pinosomes for experimental loading of antigen into the cytosol [18, 30]. This procedure allows for antigen delivery directly to the cytosol and enables a parallel and quantitative comparison of similar protein amounts delivered to distinct intracellular locations namely the cytosol versus the conventional endolysosomal pathway by isotonic fluid-phase uptake. The approach for hyperosmotic lysis based delivery of antigen to the cytosol versus exogenous delivery can thus be considered representative of biosynthetic and exogenous routes of antigen delivery. The possible presentation of residual antigen from unruptured pinosomes through the classical pathway has been formally shown not to occur, establishing this distinction between residual endosomal and truly cytosolic source of loaded antigen [18].

Based on this background, we attempted in the present study to examine whether Hsp90 contributed differentially to MHCII-restricted presentation of cytosolic and endosomal proteins. However, our data show that peptide ligands generated through processing of both endosomal and cytosolic antigen sources rely on Hsp90 for optimal presentation. The obtained results are supported by the analysis of presentation of I-Eα processing as a physiologically endogenous antigen presentation system.

Prior data from our group has demonstrated that while distinct proteolytic mechanisms support the generation of peptide ligands in the cytosol versus endosomes, antigenic peptides from both sources bind MHCII in the LAMP-1-positive endo-lysosomal compartments [21]. The requirement of Hsp90 for the presentation of both endogenous as well as internalized antigen presentation suggests two possibilities. Hsp90 may function to chaperone peptides in either subcellular location up to the point of MHCII association, and/or participate in MHCII peptide-loading complex formation. Since the endolysosomal compartment serves as a focal point for loading of peptide ligands on MHCII, we assessed the possible endosomal contribution of Hsp90 to the generation of stable MHCII heterodimers. Efficient peptide loading favors the formation of stable MHCII heterodimers resistant to disruption by chaotropic agents such as SDS and onward transit of MHCII-peptide complexes to the cell surface is determined by stable peptide binding. Enhanced levels of SDS-stable MHCII heterodimers in cell lines overexpressing Hsp90, and the dose-dependent inhibitory effect of geldana-mycin on stable dimer levels, both correlate well with a functional role for Hsp90 in effective antigen presentation through the MHCII pathway.

Bearing in mind the role of Hsp90 as a chaperone in assembly of cellular proteins, it is plausible that geldanamycin-mediated inhibition of MHCII-restricted presentation could be due to overall effects on MHCII folding and assembly. We find that both upon geldana-mycin treatment and overexpression of Hsp90, the MHCII expression patterns remain unaltered ruling out the possibility of direct effects of Hsp90 on the MHCII biosynthetic pathway as a cause for inhibitory effects of geldanamycin. Longer incubation with geldanamycin that completely abrogated both endogenous EapL presentation and SDS-stable dimer generation did not alter the MHCII expression patterns in treated cells. It seems possible that either the surface levels of CLIP loaded MHCII may be increased in geldanamycin-treated APC, or that there is increased representation of floppy dimers of MHCII as has been reported previously [38]. Furthermore, the absence of any changes in the CD80 and CD86 expression profiles excludes any nonspecific effects on stability of cell surface molecules.

The Hsp90-dependency of stable MHCII dimers on APC suggests that chaperones like Hsp90 might either directly associate with floppy dimers and facilitate formation of peptide associated compact dimers, or function to chaperone the optimal peptide for binding to MHCII. Our initial experiments in this regard suggest a direct association of Hsp90 with MHCII (unpublished observations). The sensitivity of synthetic EapS peptide presentation to geldanamycin points out that MHCII-restricted peptide loading in the endosomal compartments occurs through a process aided by Hsp90. Interestingly, this effect on peptide presentation is an MHCII-specific property of Hsp90 in the sense that presentation of exogenously added MHCI-restricted peptide is unaffected by Hsp90. A non-exclusive additional possibility could also be that Hsp90 enhances the stability of mature peptide-MHC complexes. Thus, Hsp90 appears to integrate with various aspects of the MHCII presentation pathway, and its effects on the peptide loading process may be one of the factors determining stability of peptide loaded MHCII.

Geldanamycin is reported to adversely affect T cell activation and IL-2 secretion. The use of paraformalde-hyde-fixed, inhibitor-treated APC ensures that the observed effects of geldanamycin are APC-specific and not due to other effects on T cells. That the observed inhibitory effects of geldanamycin in our system are specific to Hsp90 is borne out by retrovirus-mediated transfer of Hsp90 to APC. Cells overexpressing Hsp90 exhibit a phenotype complementary to that observed following treatment with geldanamycin. We have ruled out retroviral transduction as the cause for observed enhanced antigen presentation phenotype by the use of control APC transduced with eGFP-carrying retrovirus. Further, the sensitivity of the phenotype to inhibition by geldanamycin provides another level of confirmation for the specific role of Hsp90.

We find that unlike the uniform dependence of the MHCII-restricted presentation pathway on Hsp90 in macrophage and B cell lineages, presentation in the context of MHCI in B cells versus macrophages is distinct in terms of Hsp90 requirements. A potential explanation for this lineage-specific effect in which B cells require Hsp90 for MHCI-restricted presentation while macro-phages do not, could be related to distinct functional attributes of immuno-proteasomes in B cells versus macrophages, although this issue needs to be addressed in further detail.

Our data reveal a strong dependence of MHCII-restricted presentation of internalized exogenous antigen on Hsp90 function. This seems intriguing in context of the cytosolic localization of Hsp90. Although the precise sub-cellular location of Hsp90 with regard to MHCII processing compartments is not clearly under-stood, recent findings report the presence of Hsp90 on the cell surface in monocytes, macrophages and EBV transformed B cells ([46–48] and our unpublished results). Experimental evidence supports existence of chaperones and co-chaperones such as Hsp90, Hsc70 on the lysosomal membrane participating in lysosomal proteolytic pathways such as chaperone-mediated autophagy [49, 50]. Thus, it has been proposed that cytosolic chaperones can enter the lysosomal lumen. It seems likely that cytosolic proteins such as Hsp90 and associated ligands might access endolysosomal compartments either during antigen internalization and/or by autophagy. Another possibility is a potential role of Hsp90 in sorting of peptide-MHCII complexes to MHCII-rich multivesicular compartments in association with accessory cytosolic signaling proteins. We are currently investigating these possibilities.

**Figure 6:**
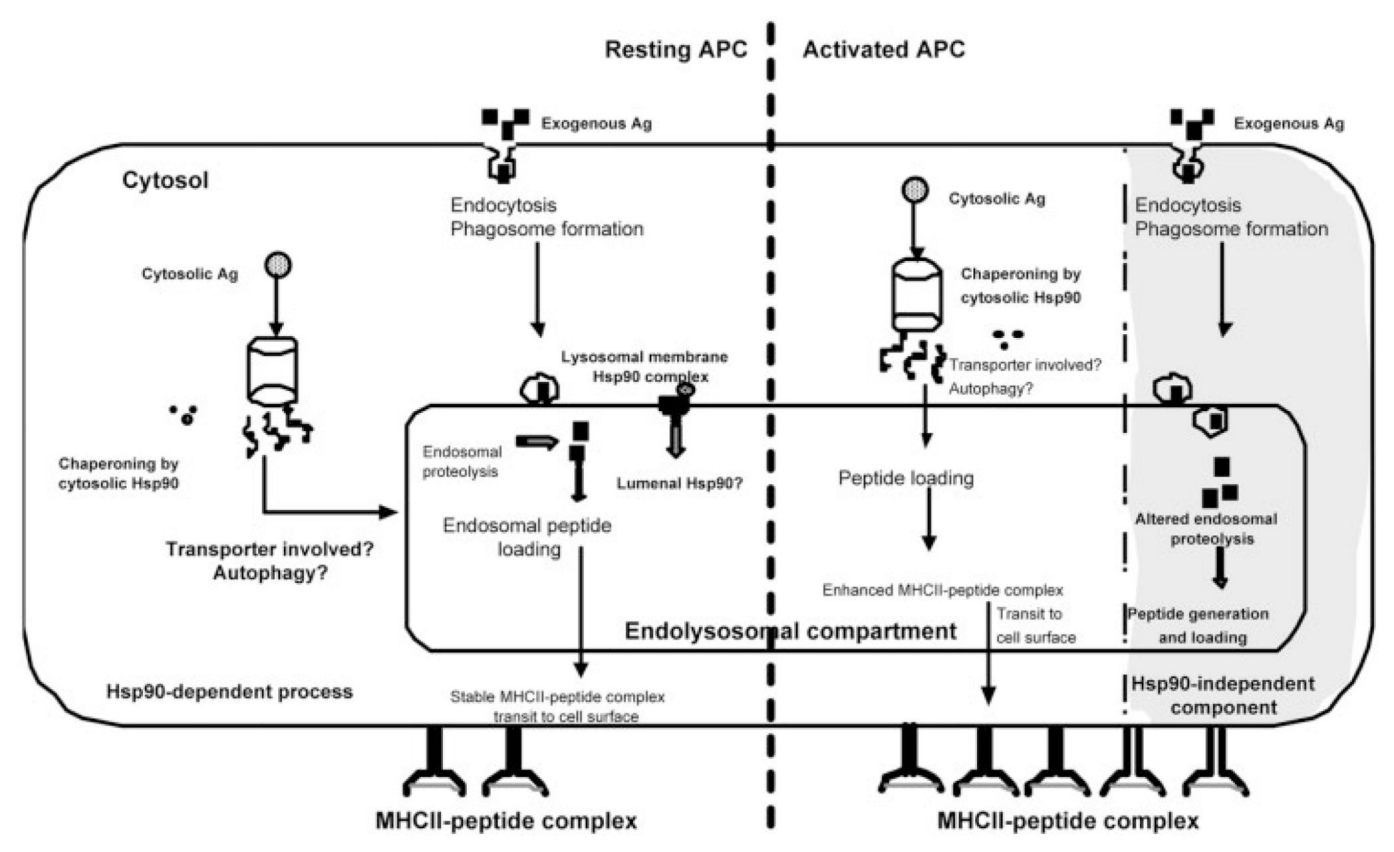
Model for the role of Hsp 90 in MHCII-restricted antigen presentation pathways in resting versus activated APC. Endogenous/cytosolic antigen presentation is reliant on Hsp90 function, irrespective of the activation status of the APC. IFN-γ-mediated activation of APC (shaded region) renders the ability to bypass this essential requirement of Hsp90 function in the exogenous antigen presentation (see text for details).

Analysis of the presentation phenotype in activated macrophage APC reveals that IFN-γ activation enhances MHCII-restricted presentation of both endogenous as well as exogenous antigens. This is unrelated to any direct effects of IFN-γ on Hsp90 induction, since we find no changes in Hsp90 expression in activated APC (data not shown and [31]). However, the presentation of endogenous processing-derived EapL versus exogenous OVA presentation by IFN-γ-activated macrophage APC exhibits a difference in sensitivity to geldanamycin. This is not observed in the case of LPS-mediated APC activation. The role for Hsp90 function in the MHCII-mediated presentation pathway thus seems to have two components. Our data present an essential Hsp90 function in presentation of peptides derived from endogenous antigen sources, irrespective of the APC activation status. The requirement for Hsp90 function in the presentation of exogenous antigen reveals an apparent dichotomy in that a component of the pathway can be bypassed by IFN-γ-induced APC activation (Fig. 6). IFN-γ-induced differential regulation of endolysosomal activity is known to cause a shift in the outcome of processing events [39–41] that could be both quantitative and qualitative in terms of parameters such as extensions flanking the core peptide or conformational alterations. Such ligands may be presented in an Hsp90-independent manner. It is also possible that the loading complex is differentially modified by IFN-γ to render exogenous presentation relatively Hsp90 independent.

Taken together, our results provide evidence for intricate associations of the major cellular chaperone, Hsp90, with MHCII-restricted antigen presentation pathways and uncover a novel role for Hsp90 as a functional component in modulation of immune responses.

## Materials and methods

### Mice

The mouse strains used, C57BL/6 (H-2^b^) and B10.A(3R)(H-2^*i3*^), were bred in the small animal facility of the National Institute of Immunology (New Delhi, India) and used at 8−10 weeks of age. All experiments were performed with the approval of the Institutional Animal Ethics Committee.

### Reagents

OVA (Sigma, St. Louis, MO) was dialyzed extensively against PBS, to remove small degradation products. All inhibitors such as geldanamycin, cytochalasin B and concanamycin A were obtained from Sigma. Geldanamycin was reconstituted in DMSO and used at a final concentration of 7.5 μM in the assays shown. Cytochalasin B was used at a final concentration of 30 μM [32] and concanamycin A at 50−100 nM [33]. The concentration of inhibitors for use in assays was determined by titrations and earlier reports. Peptides corresponding to I-Eα (52-68), EapL and I-Eα (52-66), EapS were synthesized and purified as described elsewhere [51].

### Cell lines

T cell lines used were the MHCI (H-2K^b^)-restricted T cell line B3Z specific for the OVA-derived peptide, SIINFEKL, the MHCII (H2-A^b^)-restricted OVA-specific T cell hybridoma, 13.8 [18], and the I-A^b^ restricted I-Eα (56−72) peptide (Eap)-specific T cell hybridoma 1H3.1, which recognizes both endogenous and exogenous forms of Eap [18]. The H-2^b^ APC lines used were a B cell line, LB27.4, and a monocytic line, BMC-2. The mA) used were anti-H2-D^b^, K^b^ mAb Y-3, and the anti-H2-A^b^ mAb Y-3P.

### Cytosolic antigen delivery by osmotic lysis of pinosomes

Loading of proteins into the cytosol was done using osmotic lysis of pinosomes as described [18, 30]. Briefly, APC were incubated in 1 mL hypertonic serum-free Dulbecco’s modified essential medium (DMEM) containing 0.5 M sucrose, 10% polyethylene glycol (PEG) 1000, 10 mM HEPES and the antigen (protein at 10 mg/mL and peptide at 3 μg/mL) for 10 min at 37°C. The volume was then made up to 10 mL, with hypotonic DMEM (60% DMEM), followed by an additional incubation for 2 min and washing in isotonic, serum-free medium. Exogenous loading of antigen was done similarly using isotonic medium for all steps. Cells were then incubated at 37°C for 3 h to allow antigen processing prior to being fixed with 0.5% paraformaldehyde and used as APC for T cell stimulation assays.

### Antigen-presentation assays

Peritoneal exudates-derived plastic-adherent cells from mice administered thioglycollate broth i.p. were used as macro-phage APC. Resting macrophage APC were isolated by plastic adherence from PBS washes of normal mouse peritoneal cavity. The H-2^b^ APC B cell line, LB27.4, and the monocytic line, BMC-2, were also used as APC where indicated. APC were pre-incubated for 1 h with 7.5 μM geldanamycin. The drug was dissolved in DMSO as a stock solution and used at a final maximal DMSO concentration of 0.1%. For inhibition of endogenous antigen presentation APC were incubated over-night with 5 μM geldanamycin. Cells were washed and loaded with antigen either exogenously or cytosolically as described. Fixed APC were titrated starting from 3 × 10^5^ cells/well, in a total culture volume of 200 μL/well DMEM with 10% FCS (Biological Industries, Rehovot, Israel), antibiotics, L-gluta-mine and 0.05 mM 2-ME in 96-well flat-bottom plates (Nunc, USA). Responder T cell lines were added at 1 x 10^5^ cells/well and the plates were incubated for 24−36 h prior to analysis for IL-2.

For stimulation assays of the 1H3.1 T cell line, IL-2 levels in the culture supernatant were measured using commercial enzyme immunoassay systems for IL-2, according to the manufacturer’s instructions (R& D Systems, Minneapolis, USA). Both the B3Z and 13.8 T cell lines carry the *lacZ* gene under a regulatory element from the IL-2 promoter [52]. Thus, for activation of both 13.8 and B3Z, β-galactosidase production was quantitated by a lacZ assay as detailed elsewhere [53]. Briefly, intracellular production of β-galactosidase was detected following 24−36 h of culture using a substrate solution containing the chromogenic substrate, chlorophenol red-β-D-galactopyranoside (CPRG; Roche Diagnostics, Mannheim, Germany) in PBS, pH 7.4, containing 0.125% NP-40, 100 mM 2-ME, 9 mM MgCl_2_. Activation was monitored by measuring absorbance at 570 nm.

### Retrovirus-mediated gene transfer

A cDNA encoding human Hsp90 [31] cloned at the XhoI site of the retroviral vector pMSCV-puro (Clontech, San Jose, CA) was a kind gift from Dr. H. Udono (Department of Molecular Medicine, Nagasaki University School of Medicine, Nagasaki, Japan). The construct was transfected into the packaging cell line, PT67, using Fugene 6 (Roche Molecular Biochemicals) according to the manufacturer’s instructions. Cells were subjected to selection at 5 μg/mL of puromycin 24 h post-transfection. The puromycin-resistant virus-producing PT67 cells were expanded and maintained in puromycin. For control transductions, the *eGFP* gene was cloned in the BglII/NotI site of the retroviral vector, pLNCX2 (Clontech, San Jose, CA) and transductants selected for G418-resistance. Culture super-natants from resistant clones were used as a source of virus for transduction.

Retroviral transduction with Hsp90 virus-containing culture supernatants for both LB 27.4 and BMC-2 was carried out in the presence of 6 μg/mL of polybrene, and cells were subjected to puromycin selection 24 h post-transduction. Stable clones identified following 2−3 weeks of culture were maintained in the presence of puromycin. Similar transductions were also performed with eGFP-bearing retrovirus and transductants similarly subjected to G418 selection. Prior to use in antigen-presentation assays, cells were washed extensively, cultured overnight in the absence of selection and subsequently used as APC for presentation to T cells. For flow cytometric analysis, the cells from selection cultures were similarly washed and stained as outlined below.

### Flow cytometry

APC were incubated with mAb culture supernatants for 1 h on ice, followed by washes in cold staining buffer (PBS containing 0.5% BSA with 0.01% sodium azide). The secondary Ab used was PE-conjugated goat anti-mouse Fc-specific, IgG F(ab)_2_ (BD PharMingen, San Jose, CA). Flow cytometric data were collected on a BD LSR flow cytometer (BD) and analyzed with FlowJo software (Treestar, San Jose, CA). For staining Hsp90, a mouse mAb raised against the C-terminal (586−732) peptide of human Hsp90 and reactive against both mouse and human Hsp90 (BD PharMingen, CA) was used. For permea-bilization, cells were fixed and permeabilized with permeabilization buffer (BD Biosciences) for 15 min on ice and washed extensively in Perm Wash buffer (BD Biosciences) according to the manufacturer’s instructions. Incubation with primary and secondary antibodies was subsequently carried out in Perm Wash buffer and as described above.

### SDS-PAGE and Western blot analyses of MHCII dimers

Whole-cell lysates of variously treated APC were made after washing the cells once in ice-cold PBS. The cell pellets were resuspended at 4°C in 2-ME-free lysis buffer (1% NP-40, 1 mM EDTA, 150 mM NaCl, 20 mM Tris.Cl, pH: 8.0, 1 mM MgCl_2_ containing 10 μg/mL leupeptin and aprotinin). Cell lysates from an equivalent of one million cells each were resolved on 10% SDS-PAGE and transferred to nitrocellulose membranes for Western blot analysis. Membranes were incubated with antibody containing culture supernatant overnight at 4°C. After six washes at room temperature, horseradish peroxidase (HRP)-labeled goat-anti-mouse IgG was added for 1 h at room temperature. The membranes were again washed and developed using di-aminobenzidine/hydrogen peroxide, DAB/H_2_O_2_-containing substrate solution. For detecting actin, rabbit polyclonal anti-actin antibody (Santa Cruz Biotech, Santa Cruz, CA) followed by HRP-conjugated donkey-antirabbit-IgG was used. Hsp90 was detected using the mouse mAb mentioned above.

### IFN-γ and LPS stimulation assays

In assays involving IFN-γ stimulation, 72-h thioglycollate-elicited peritoneal exudates cells (PEC) were harvested and rested overnight. Rested adherent PEC were either treated with IFN-γ at 10 U/mL for 48 h or left untreated in complete medium. In the last 24 h of incubation, geldanamycin was added at a concentration of 5 μM. The cells were harvested, washed and then pulsed with OVA in isotonic medium as described above at a concentration of 3 mg/mL. The APC were similarly incubated for a further period of 3 h to allow antigen processing in the presence or absence of IFN-γ and/or geldanamycin. The Tcell stimulation assays were subsequently set up as described earlier. LPS stimulation was similarly performed with LPS at a concentration of 3 μg/mL.
